# Generation of ssDNA aptamers as diagnostic tool for Newcastle avian virus

**DOI:** 10.1371/journal.pone.0237253

**Published:** 2020-08-13

**Authors:** Boutheina Marnissi, Masood Kamali-Moghaddam, Abdeljelil Ghram, Issam Hmila

**Affiliations:** 1 Laboratory of Epidemiology and Veterinary Microbiology, Institute Pasteur of Tunis, University Tunis El Manar, Tunis, Tunisia; 2 Department of Immunology, Genetics and Pathology, Science for Life Laboratory, Uppsala University, Uppsala, Sweden; Universidade de Sao Paulo Instituto de Quimica, BRAZIL

## Abstract

Aptamers are short single-stranded DNA (ssDNA), RNA or synthetic XNA molecules, which are used as a class of affinity binders recognizing target molecules with a very high affinity and specificity. The aim of this study was to generate and characterize ssDNA aptamers for the detection of Newcastle disease virus (NDV). These aptamers were selected using systematic evolution of ligands by exponential enrichment (SELEX) in combination with quantitative high-throughput DNA sequencing. After three rounds of selections, a highly enriched ssDNA pool was sequenced, and the results were analyzed using FASTAptamer Toolkit. Sequencing reads were sorted by copy numbers and clustered into groups, according to their sequence homology. Top aptameric sequences were used to develop a sandwich enzymatic linked aptamer assay (ELAA) for rapid and sensitive detection of NDV in farm samples. The selected aptamers have an affinity within the nanomolar range, and a high specificity with no cross-reactivity towards other avian viruses. Following optimization of the sandwich ELAA method, the results demonstrated that both selected aptamers Apt_NDV01 and Apt_NDV03 with dissociation constant values of 31 nM and 78.1 nM, respectively, showed the highest specificity and affinity for NDV detection. The ELAA results were verified by quantitative real-time PCR, demonstrating strong concordance, and showing outstanding accuracy for detection of NDV in field sample. In summary, combination of SELEX with high-throughput DNA sequencing allowed rapid screening and selection of aptamers. The selected aptamers allowed recognition of NDV with high affinities. This is the first report that uses a validated sandwich ELAA for rapid and specific detection of NDV in poultry samples.

## Introduction

Newcastle disease (ND) is an acute and highly contagious avian disease, which causes heavy economical losses to poultry industry, worldwide [1]. The causative agents are virulent strains of Avian Paramyxovirus type 1 (APMV1). In regard to their pathogenicity for chickens, ND virus (NDV) strains are divided into three pathotypes; velogenic (highly fatal), mesogenic (intermediate virulence) and lentogenic strains (low virulence). Some members of the latter, such as LaSota and V4 subtypes are used as live vaccines [2].

The virus belongs to the genus *Avulavirus* of the family *Paramyxoviridae*, in the order of *Paramyxovirinae*. The viral genome is a non-segmented, negative-sense and single-stranded RNA of approximately 15.2 kilobases (kb), encoding six main proteins; nucleoprotein (N), phosphoprotein (P), matrix protein (M), large polymerase protein (L), hemagglutinin–neuraminidase (HN) glycoproteins, and fusion protein (F) [1, 3]. The glycoproteins HN and the F protein are located on the surface of the viral membrane, and induce neutralization antibody production. They enable the virus to attach to sialic acid-containing receptors on the host cells, and are responsible for the neuraminidase activity [4]. The F protein is responsible for the fusion of the viral envelope with the cellular plasma membrane [5]. These two proteins are also considered as biomarkers for NDV.

The NDV infects wild birds and poultry species. It has been reported as a major problem for the poultry sector in Latin America (Mexico, Colombia, Venezuela, and Peru) [6], in Iran [7] and several other countries, worldwide. However, data on the prevalence of the disease are insufficient in Tunisia, and the virus continues to affect the profitability of poultry production. Biosecurity measures and vaccination programs should be enforced to ensure better control and mitigate virus incidence, avoiding the emergence of more pathogenic strains. To monitor this major viral disease and to avoid health crisis in poultry, the development of sensitive assays for early detection, enabling characterization of circulating viruses is needed and will have great economical and epidemiological significances. Appropriate diagnosis is the key factor for better handling viral diseases. Nevertheless, many avian viral diseases are difficult to distinguish, especially at the disease onset with a set of similar and nonspecific clinical symptoms.

To develop efficient tools for the diagnostic of these major viral diseases, the potential of aptamers was explored as an affinity binder for NDV. Aptamers are short single-stranded DNA (ssDNA), RNA, synthetic XNA oligonucleotides or peptide molecules, which can recognize their targets with high affinity and specificity [8]. Their production, using an experimental procedure termed SELEX (Systematic Evolution of Ligands by Exponential Enrichment), is easy and inexpensive [9]. Since their discovery, the aptamers have rapidly emerged as a key factor in several biological processes such as diagnostic, therapy, drug discovery, food science, and drug delivery [10]. Several studies have been conducted on the aptamers’ potential use for the diagnostic of different diseases including cancer and infectious diseases [11, 12]. Recently, aptamers against influenza virus have been demonstrated as an potential alternative tool to antibodies with equal or greater affinity to HA antigen [13, 14]. New aptamers against H9N2 influenza subtype have been characterized in our laboratory and have been used to develop a highly sensitive real-time immuno-PCR-based test [15].

Similarly, characterization of aptamers against other viruses including respiratory syncytial virus (RSV), severe acute respiratory syndrome virus (SARS) and measles virus have already been reported [16]. However, to date there is no reports on aptamers developed for avian virus detection. The present study reports on a rapid and an efficient approach for the selection and characterization of ssDNA aptamers against NDV. The generated aptamers were then tested for their affinity and specificity to efficiently detect the viral antigens in farm samples.

## Materials and methods

### Ethics statement

No vertebrate animals, embryos or tissues have been involved in the present work.

For validation of the generated aptamers, the leftover chicken samples received for routine diagnostics, were used. No further ethical approval was obtained as no laboratory animals were used in this study.

### Virus strains and field samples

LaSota vaccine strain of NDV, live H120 vaccine strain of infectious bronchitis virus (IBV), live IBDV vaccine strain of Gumboro virus, attenuated live 1133 strain of avian reovirus and live avian influenza Tunisian isolate A/CK/TUN/145/12 subtype H9N2 were used for the specificity test. The study was performed on a total of 27 poultry samples including tracheal (ET) and cloacal swabs (EC) and internal organs consisting of allantois (A), kidneys (K), lung (L), liver (Li) and trachea (T) collected from chickens with suspected NDV infection. The field samples were collected from eight farms located in the following provinces: Bizerte, Nabeul, Ben Arous, Sidi Bouzid, Beja, Ariana, Sfax and Jendouba, and were sent by veterinarians to the diagnostic laboratory at Institute Pasteur of Tunis for analysis, in the frame of their routine follow-up/monitoring of poultry farms, or in case of reporting a disease suspicion, especially for the diagnostic of NDV and avian influenza, which are under continuous surveillance in Tunisia. Samples information including the region, the veterinary or/and farm or/and company name as well as type of production and age of poultry are summarized in S1 Table in S1 File.

### Systematic evolution of ligands by exponential enrichment (SELEX)

The NDV-bound ssDNA aptamers were selected from a ssDNA library, using a slightly modified SELEX protocol reported by Hmila et al. and Arnold et al. [15, 17]. The random DNA library (WAP40m) consisted of 80 nucleotides (nt) containing a 40 nt randomized central region flanked by two adaptor sequence regions each of 20 (Integrated DNA Technologies, Inc., Coralville, IA). The library sequence– 5’-AGTGCAAGCAGTATTCGGTC–(N)_40_-TAAAGCTGATGCGTGATGCC-3’–was used as reported by Lamont et al. [18]. Before selection, the ssDNA library was denatured by heating at 95°C for 5 min then cooled on ice for 10 min to prevent inter-strand base pairing. SELEX was initiated with the immobilization of 100 μL of diluted LaSota vaccine strain in 96-well microtiter plate overnight at 4°C. The plate was then dry blotted, and 100 μL of the single-stranded aptamer DNA library (10 μM) was mixed with the virus and incubated for 1 h with gentle shaking. The wells were dry blotted, and then 100 μL of phosphate-buffered saline (PBS; pH 7.2; 150 mM NaCl + 5mM MgCl2) was added and incubated for 5 min at room temperature (RT).

A NaCl salt gradient was used for the elution of the enriched aptamers; the wells were sequentially washed using five different concentrations of 0.5, 1.0, 1.2, 1.4, and 1.5 M NaCl, allowing gradual releasing of aptamers, starting from those with the weakest to strongest binding affinities. The molecular basis for this phenomenon is mainly related to the NaCl's ability to sever electrostatic interactions, the key interactions between the aptamers and the protein [19].

Then, 2 μL of the isolated DNA oligonucleotides from the 1.5 M elution was amplified by PCR, and was used to recognize the virus pre-immobilized on a membrane nitrocellulose. Products from the aptamer-spots were extracted and a symmetric PCR amplification was performed with an initial heating step for 5 min at 95°C followed by 25 cycles of 95°C for 30 s, 55°C for 30 s, 72°C for 30 s and a final extension step at 72°C for 7 min. The reaction mixture contained 5 μL of 10X PCR buffer, 3 μL of SELEX pool, 0.5 μL (10 μM) of each forward WP20F1 (AGT-GCA-AGC-AGT-ATT-CGG-TC) and reverse WP20R1 (TAA-AGC-TGA-TGC-GTG-ATG-CC) primers (Integrated DNA Technologies, Inc., Coralville, IA), 1 μL MgCl_2_ (50 mM; ThermoFisher Scientific), 1 μL dNTP (10 mM; ThermoFisher Scientific), 0.5 μL Taq polymerase (ThermoFisher Scientific), and the volume was adjusted to 50 μL with deionised water. An asymmetric PCR was performed using 2 μL symmetric PCR products, and an optimal ratio of forward and reverse primers of 25:1, respectively. The thermocycles were initiated with a heating step for 5 min at 95°C followed by 9 cycles of 95°C for 30 s, 63°C for 15 s, 72°C for 15 s, and a subsequent 10 cycles of 95°C for 30 s, 55°C for 15 s, 72°C for 15 s, and a final step of 72°C for 3 min. The reaction mixture was composed of 5 μL of 10X PCR buffer, 2 μL of symmetric PCR product, 1.2 μL of WP20R (1 μM), 3 μL of WP20F (10 μM), 1 μL of MgCl_2_ (50 mM), 1 μL of dNTPs (10 mM), 0.5 μL Taq polymerase in a total volume of 50 μL, adjusted with deionised water.

### High-throughput sequencing technology

The PCR product of the SELEX amplified products using WP20F1 primer was purified with the MinElute® PCR Purification Kit (Qiagen). The sequencing library was conducted using the AB Library Builder™ System (ThermoFisher Scientific) and amplified according to the Ion Xpress™ Plus and Ion Plus Library Preparation for the AB Library Builder™ System protocol. It was then purified using the Agencourt® AMPure® XP reagent (Beckman Coulter). The library size and its concentration were assessed by a Bioanalyzer High Sensitivity Chip (Agilent Technologies). The samples were pooled, followed by a template preparation on the Ion Chef™ System, using the Ion PI Hi-Q Chef Kit (ThermoFisher Scientific). The samples were then loaded on an Ion PI™ v3Chips and sequenced on the Ion Proton™ System, using the Ion PI™ Hi-Q Sequencing 200Kit chemistry (ThermoFisher Scientific).

### Bioinformatics analysis

The dataset analysis allowed identification and removing of the specific 5′- and 3′-primer binding regions in each sequence, using a command line. Subsequently, the sequences were filtered by discarding those longer than 50 nt and keeping the intern randomized regions of the original aptamer sequences. The majority of these sequences had an expected length of 40 ± 3 nt after pre-processing and filtration of the aptamer dataset.

FASTAptamer Toolkit was used following the same steps previously described [19]. First, the FASTAptamer-count package was used to rank and sort the sequences by their abundance by counting the number of times each sequence is sampled from a population, according to their copy numbers. Second, the FASTAptamer-cluster was used to align and classify the reads by sequence similarity. Finally, sorted sequences were evaluated for their affinity and specificity.

Selected ssDNA molecules were subjected to secondary structure prediction, using mfold software (http://mfold.rna.albany.edu/?q=mfold/DNA-Folding-Form).

### Affinity binding and specificity of selected aptamers

The ELAA was employed to test the binding affinity of the selected aptamers to NDV, using the following procedure. LaSota vaccine virus was diluted in PBS and 100 μL were used to coat the wells of a 96-well microtiter plate at 4°C overnight. The wells were then rinsed three times with PBS-T (10 mM PBS, pH 7.2; 0.05% Tween-20) and blocked with 200 μL/well of 5% skim milk at RT, for 1 h. Next, a volume of 100 μL/well of a 2-fold dilution serial of 5’-biotin-labeled DNA aptamers from 10,000 to 0.15 nM was added to NDV coated wells and incubated at RT for 1 h. A naïve library at 10 nM in 100 μL was used as a non-specific binding control. The wells were then washed three times with PBS-T to remove unbound aptamers. Streptavidin-horseradish peroxidase conjugate (1:5000) was added, and incubated at RT for 30 min. After five washes with PBS-T, OPD (O-Phenylene Diamine dihydrochloride) was added for visualization. Finally, the reactions were stopped by adding 50 μL/well of H_2_SO_4_ (2N), and the optical densities (OD) were recorded at 492 nm, using a microplate reader. To determine the equilibrium dissociation constants (K_d_) for the candidate aptamers, the OD value mean of a naïve library was subtracted from the OD value mean of each aptamer reading.

To evaluate their specificity for NDV, the binding of the five selected aptamers Apt_NDV01 to Apt_NDV05 were tested against the LaSota live vaccine strain of NDV, the H120 live vaccine strain of IBV, the isolate A/CK/TUN/145/12 (H9N2) isolate of IA, IBDV vaccine strain of Gomboro disease, attenuated 1133 strain of reovirus and a naïve library as negative control as described above. All the experiments were performed in triplicate.

### Sandwich ELAA test

A sandwich ELAA was carried out in Streptavidin Coated Plates (ThermoFisher Scientific), using one of the biotin-labeled Apt_NDV02, Apt_NDV03, Apt_NDV04 or Apt_NDV05 as a capture, and digoxigenin-labeled Apt_NDV01 as a reporter molecule. Each 96-well microtiter plate was coated with 100 μL of 10 nM biotin-aptamers and incubated at RT for 1 h, washed three times with 200 μL of washing buffer PBS-T (10 mM PBS, pH 7.2; 0.05% Tween-20). Next, LaSota vaccine virus was added in 100 μL PBS (pH 7.2) and incubated for 1 h at RT. Wells were then washed five times with PBS-T and 1 μM of the reporter digoxigenin-modified Apt_NDV01 diluted in 100 μL PBS was added, and the incubation was continued for an additional 1 h. Anti-digoxigenin antibody (1:2000) was added to the corresponding wells to react for 30 min, followed by three washes. Finally, OPD was added before stopping the reaction by adding H_2_SO_4_ (2N). The absorbance was measured at 492 nm and the results of each combination were calculated as the mean ± SD from three experiments.

### Establishing the sandwich ELAA to detect NDV in field samples

A total of 27 samples from poultry suspected to be infected with NDV, including tracheal (ET) and cloacal swabs (EC) and internal organs consisting of allantois (A), kidneys (K), lung (L), liver (Li) and trachea (T) were analyzed. The samples were assessed for NDV using the sandwich ELAA, in streptavidin coated microtiter plates. First, 100 μL of 10 nM Biotin-Apt_NDV03 were incubated at RT. After 1 h incubation, the wells were washed three times with 200 μL of PBS-T, and 1 μL of each farm sample diluted in 100 μL PBS was added into each well, and incubated for 1 h at RT. After three washes with PBS-T, 100 μL of 10 nM digoxigenin-labeled Apt_NDV01 was added, incubated for 1 h at RT and the plate was washed five times. The anti-digoxigenin antibody (1:2000) was then added to each well and incubated for 30 min. After three washes, a solution of OPD was added before the reaction was stopped by H_2_SO_4_ (2N), and the absorbance were recorded at 492 nm.

### One step real-time PCR

One step qRT-PCR was conducted in a total volume of 12 μL, using AgPath-ID™ One-Step RT-PCR Kit (Applied Biosystems TM) by mixing 7.5 μL 2X RT-PCR Buffer, 0.6 μL of 25X RT-PCR Enzyme Mix, 2 μL RNA template, 0.3 μM of each forward (Fr-5′-TYGAGGGACTTGAAYGTTGAC-3’) and reverse primer (Rv-5′-CCTGAGGAGAGGCATTTGCTA-3’) specific to polymerase (M) gene [20] and 0.2 μM TaqMan™ probe (P: 5′ FAM–TTCTCTAGCAGTGGGACAGCCTGC–TAMRA-3’). The reactions were conducted by heating the mixture at 45°C for 10 min followed by 95°C for 10 min and 45 cycles of 95°C for 15 s and 60°C for 45 s. A negative control lacking the RNA template was included in each PCR run.

### Limit of detection of qRT-PCR and sandwich ELAA

LaSota vaccine strain titrating 10^6^ EID_50_/ml, was used to determine the limit of detection (LOD) of qRT-PCR. A 100 μL volume of the vaccine was treated to extract the viral RNA, which was used to prepare a two-fold serial dilution, and then the qRT-PCR was performed as described above. The data was calculated to determine the LOD of the assay.

Also, to determine the LOD of the sandwich ELAA, an aliquot of 10^6^ EID_50_/mL of LaSota vaccine strain was used to prepare two-fold serial dilutions from which 100 μL was added to each well of a microtiter plate pre-coated with 100 μL of 10 nM biotinylated Apt_NDV03. The plate was incubated at RT for 1 h, the wells were washed three times with 200 μL of washing buffer PBS-T, and then, 1 μM digoxigenin-Apt_NDV01, diluted in 100 μL PBS, was added to each well. After 1 h incubation at RT, anti-digoxigenin antibody (1:2000) was added and the incubation continued for an additional 30 min. The wells were then washed once with PBS-T, the OPD was added and the optical densities were recorded at 492 nm.

### Statistical analysis

Statistical significance of the sandwich ELAA and qRT-PCR results was determined by one-way ANOVA test, using Simple Inter-active Statistical Analysis (SISA) online tool (http://www.quantitativeskills.com/sisa/index.htm), and defined as significantly different if < 0.01. For more relevant statistical significance analysis, a 95% confidence interval (CI) of the mean, a range with an upper and lower number calculated from a sample was determined by one-way ANOVA test.

The mean and the standard deviation (SD) and the coefficient of variation percentage (CV%) of optical densities (OD 492 nm) for sandwich ELAA and the threshold cycle (Ct) of qRT-PCR were further analyzed, using Excel software.

To measure the relative variability between triplicates of one sample, CV% was calculated as CV% = (STDEV of the sample triplicates /Mean of the sample triplicates) *100 and defined as significantly different if CV% < 20%.

StatPlus Pro version 5.9.8 was used to calculate the LOD, the LLOQ, the ULOQ, the MDD and the sensitivities of both methods.

The LOD is the lowest analyte concentration that can be accurately differentiated from the background and at which its detection is feasible. A conventional and standard LOD estimation method consists on the measurement of replicates of background or blank sample, the determination of the mean values and SD and the calculation of the LOD as the mean + 2 SD [21]. The LOD was calculated as LOD = background signal + (3 x SD_mean_), where the background signal corresponds to the mean value of three negative control samples; the SD mean being the standard deviation of those values. Additionally, the lower limit of quantification (LLOQ) is the lowest concentration at which the analyte can be accurately identified and at which certain predefined bias and imprecision targets are achieved. Furthermore, the LLOQ value may be comparable to the LOD or at a higher concentration. LLOQ were determined as LLOQ = LOD + (10 x SD_background_). The upper limits of quantification (ULOQ) was determined as ULOQ = f (x–(3 x SD_x_), whereas the minimal detectable dose (MDD) was determined as MDD = 2 x SD_background mean_.

## Results

### Aptamer selection

DNA-aptamers against NDV were generated using the SELEX protocol described by Hmila et al. [15]. Briefly, the LaSota virus vaccine was incubated with a randomized DNA pool and washed. The output of the SELEX was amplified by symmetric (S1 Fig in S1 File) and asymmetric PCR, and the final PCR products were incubated with the virus immobilized on a nitrocellulose membrane, using an immune-blot test to monitor the enrichment of target-binding aptamers. The products from the aptamer-immobilized spots were subsequently used for the next rounds of selection. After three rounds of SELEX, an increased fluorescence signal was observed (S2 Fig in S1 File). Then the DNA pool was sequenced using high-throughput sequencing.

### High-throughput DNA-aptamers sequencing technology

The DNA sequence pools (S2 Table in S1 File) were analyzed using FASTAptamer software. The pre-processed sequences were filtered and then analyzed by FASTAptamer-count to sort the reads based on their abundance, which is normalized for reads per million (RPM) and ranked according to the decreasing abundance (S3 Table in S1 File). The results showed that the most abundant sequence was sampled 7,620 times, corresponding to 1,362.29 RPM. FASTAptamer-count output was used as FASTAptamer-cluster input. Using the command”FASTAptamer_cluster,” closely-related sequences were grouped into clusters (S4 Table in S1 File). As shown in Table 1, each generated random sequence was sorted by its rank, reads, RPM, cluster number, rank within that cluster and Levenshtein edit distance that reflects the number of insertions, substitutions, or deletions between sequences. From the analyzed pool, the first five sequences with high RPM were selected for further characterization.

**Table 1:** Sequences sorted from FASTAptamer-cluster.

Sequence (5’ → 3’)	Rank of sequence	Reads	RPM	Cluster number	Rank of sequence within cluster	Levenshtein edit distance
**GGGGTCTTGCAGGTCCCGTAGGAGGGGCCATTGGAGTGGGG**	1	7620	1362.29	1	1	0
**GGGGTCTTGCAGGTCCCGTAGGAGGGGCCATTGGAGTGGG**	27	613	103.59	1	2	1
**AACTTATCGGAGCGTGATTTCCGTCTCGCCGCTTTCCTTT**	2	4235	757.13	2	1	0
**AACTTATCGGAGCGTGATTTCCGTCTCGCCGCTTTCCCTT**	47	316	56.49	2	2	1
**CGATGGAGGACCTCCGGTTTACCGTGTCGTTTTACTCTTG**	3	3262	583.17	3	1	0
**CCTCGCTATGATGGAGTGCGTTTAGATCAGGGAACGGGTT**	4	2769	495.04	4	1	0
**CCTACGTTGGAGTGGGGTTTGCGCAGGCCGTTCTTTCCAA**	5	2657	475.01	5	1	0

### Binding affinity of selected aptamers

**Fig 1:**
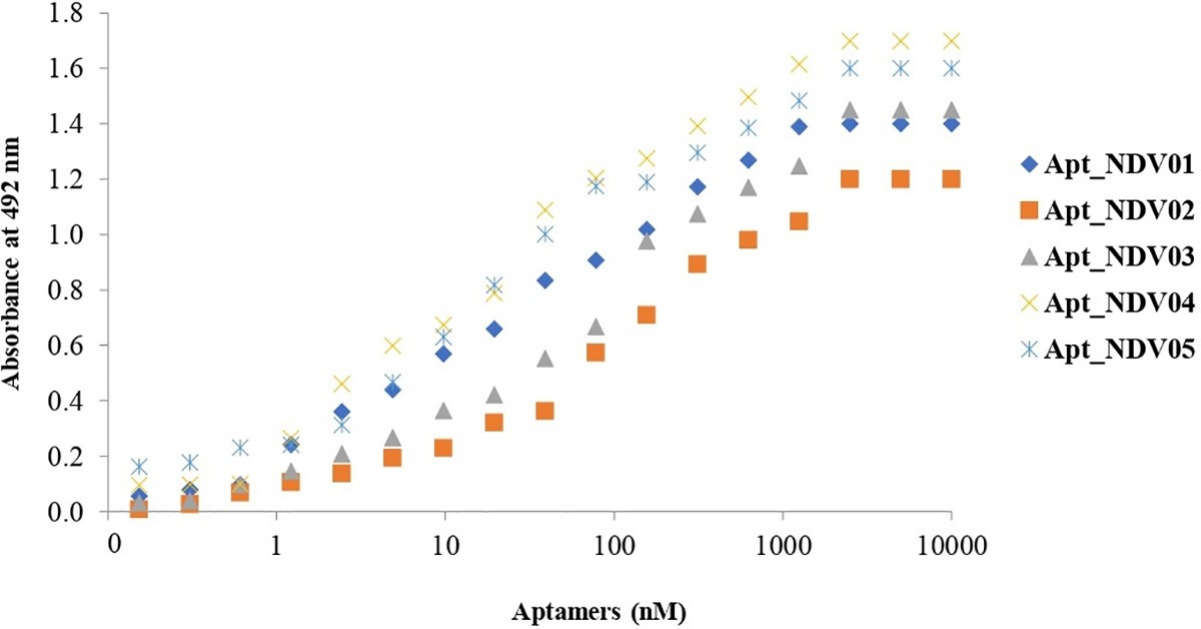
Dissociation constants (K_d_) at the lowest nanomolar range for the five selected aptamers. To calculate the K_d_s for Biotin-Apts_NDV01-05, various concentrations of aptamers were incubated with LaSota vaccine virus immobilized in wells of a microtiter plate. The absorbance at 492 nm were collected, and the K_d_ values were then calculated from the ELAA data on a nonlinear regression equation.

To evaluate the binding affinity of selected aptamers to NDV, the virus was incubated with increasing concentrations of biotin-labelled aptamers, and subsequently analyzed by ELAA, based on a nonlinear regression equation. The results showed that the five selected random sequences of aptamers have affinities at the lowest nanomolar range, and the aptamers Apt_NDV01 and Apt_NDV04 demonstrated the highest affinities (Fig 1 and Table 2).

**Table 2:** Binding affinities of the five selected aptamers.

Aptamer	Aptamer sequence (5’-3’)	K_d_ (nM)	SD
Apt_NDV01	GGGGTCTTGCAGGTCCCGTAGGAGGGGCCATTGGAGTGGGG	31	± 3.39
Apt_NDV02	AACTTATCGGAGCGTGATTTCCGTCTCGCCGCTTTCCTTT	85.1	± 2.06
Apt_NDV03	CGATGGAGGACCTCCGGTTTACCGTGTCGTTTTACTCTTG	78.1	± 3.33
Apt_NDV04	CCTCGCTATGATGGAGTGCGTTTAGATCAGGGAACGGGTT	24.3	± 3.37
Apt_NDV05	CCTACGTTGGAGTGGGGTTTGCGCAGGCCGTTCTTTCCAA	47.9	± 4.29

### Binding specificity of selected aptamers

**Fig 2:**
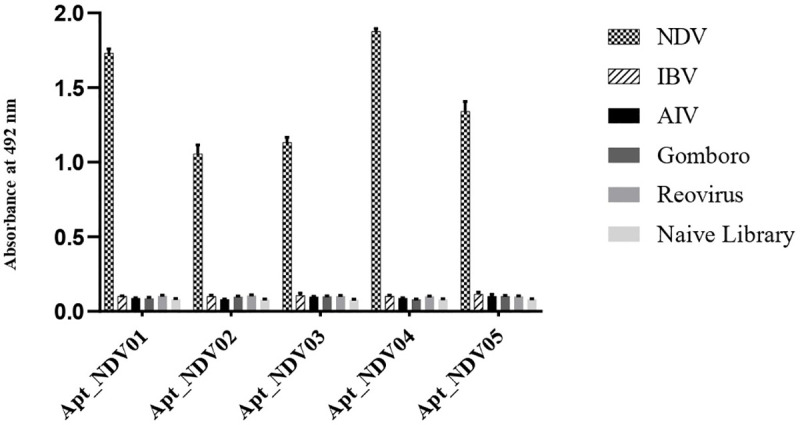
Highly specific aptamers against NDV. The specificity of five aptamers with the highest affinity for NDV were tested using ELAA to detect either vaccine strain of NDV-LaSota, H120—IBV, IBDV- Gomboro, 1133 reovirus and avian influenza-H9N2. A naïve library was used as a negative control. The test was performed in triplicate. P-values for Apt_NDV01-05 were < 0.01, calculated using one-way ANOVA test.

The specificity of the five selected aptamers, Apt_NDV01-05, against NDV was evaluated by testing their affinities against various avian viruses besides NDV-LaSota vaccine strain, including H120—IBV, IBDV- Gomboro, 1133 reovirus, avian influenza-H9N2, and a naïve library which was used as a negative control. All five aptamers showed high specificity for NDV, while no affinity for the other virus strains was observed (p-values < 0.01; Fig 2).

### Sandwich Enzyme-Linked Aptamer Assay (ELAA)

**Fig 3:**
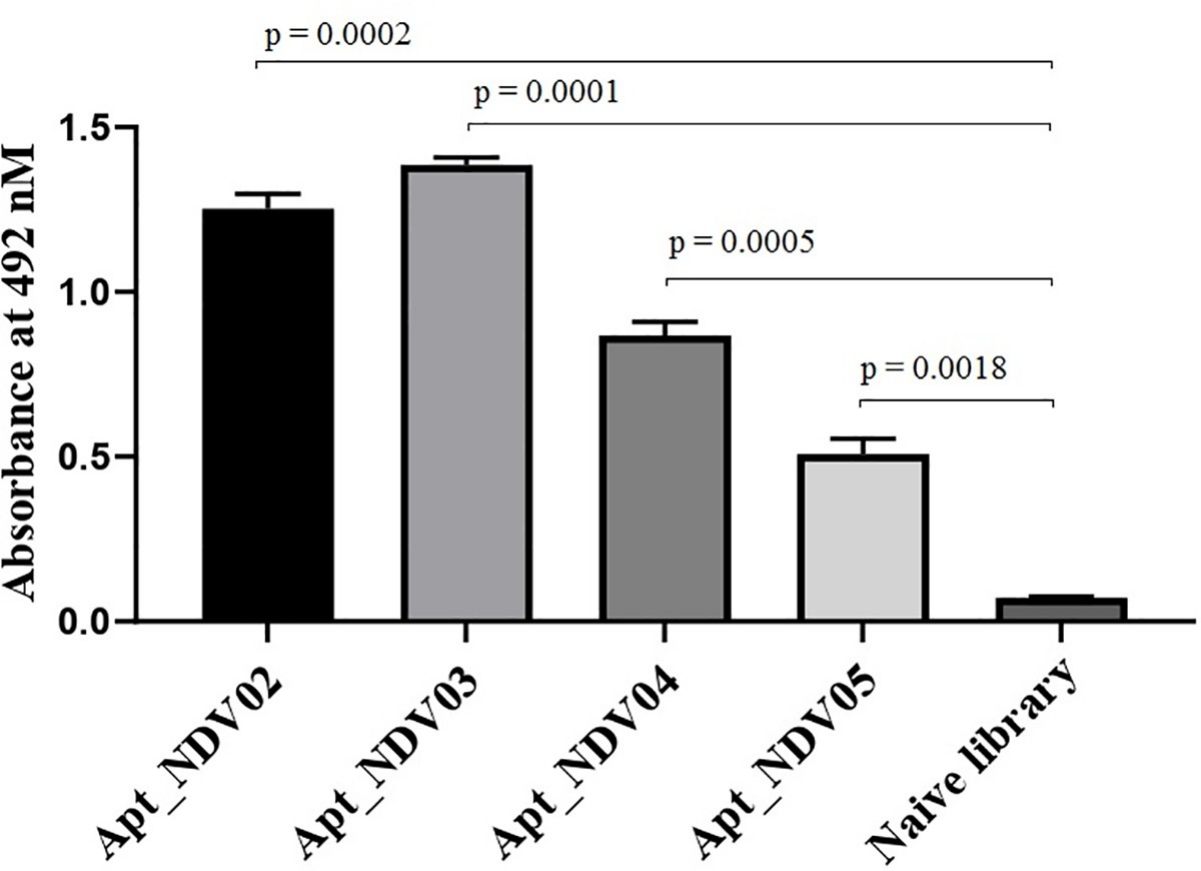
Combined aptamers for a highly efficient sandwich ELAA. Biotin-labeled Apt_NDV02, Apt_NDV03, Apt_NDV04 and Apt_NDV05 were used as capture binders, while digoxigenin-labeled Apt_NDV01 was used as a reporter in a sandwich ELAA. The recorded absorbance values at 492 nm showed that Apt_NDV01/Apt_NDV03 combination is more effective compared to the other combinations. The structure of both Apt_NDV01 and Apt_NDV03 aptamers were predicted by means of free-energy minimization algorithm using the mfold tool, available at http://unafold.rna.albany.edu/?q=mfold (S4 Fig in S1 File).

The performance of the Apt_NDV01, in combination with the other four selected aptamers, was tested using the ELAA. The results demonstrated that the Apt_NDV01 in combination with Apt_NDV03 was the most effective, closely followed by the Apt_NDV01/Apt_NDV02 combination as compared to Apt_NDV01/Apt_NDV05, which shows lower interaction with the antigen; no detectable interaction with the negative control was observed (Fig 3). A combination of Dig-Apt_NDV01 and Biotin-Apt_NDV03 was selected for their higher specificity and capture affinity for the detection of NDV in farm samples (S3 Fig in S1 File).

To compare the sensitivity as well as the performance of both sandwich ELAA and qRT-PCR methods, the optical densities (OD at 492 nm) for sandwich ELAA and the threshold cycle (Ct) of qRT-PCR were used to determine the LOD, the LLOQ, the ULOQ, the MDD and the sensitivities of the both methods (S5 Fig in S1 File). As expected, the dynamic range for qRT-PCR was greater than that for sandwich ELAA, which also demonstrates better LOD, LLOQ, ULOD and MDD. The analytical characteristics of both assays are summarized in (Table 3).

**Table 3:** Comparison of LOD, LLOQ, ULOD, MDD and dynamic range between the sandwich ELAA and the qRT-PCR for the detection of NDV.

	Sandwich ELAA	qRT-PCR
LOD (EID_50_/ml)	1.2	0.6
LLOQ (EID_50_/ml)	20	0.5
ULOD (EID_50_/ml)	7^3^	10^3^
MDD (EID_50_/ml)	1	0.2
Dynamic Range	10^3^	10^5^

### Detection of NDV in farm samples

To evaluate the ability of the selected aptamers to detect NDV in farm samples, 27 samples collected from suspected chickens were analyzed by the developed sandwich ELAA, using the Apt_NDV03 as a capture and the Apt_NDV01 as a reporter affinity binder. The results were compared to those obtained by qRT-PCR as the golden standard method.

Out of the 27 analyzed farm samples, 15 were revealed positive by the ELAA-based method, which were confirmed by the standard qRT-PCR method, indicating the good accuracy of the sandwich ELAA test (Table 4).

**Table 4:** Cross-tabulation between Sandwich ELAA and qRT-PCR. Results of the diagnosis of NDV in tracheal (ET) and cloacal swabs (EC) and internal organs, consisting of allantois (A), kidneys (K), lung (L), liver (Li) and trachea (T) collected from chickens with suspected NDV infection. Results of sandwich ELAA and qRT-PCR are reported as positive or negative for each sample.

Sandwich ELAA Results (OD)								qRT-PCR Results (Ct)						
Samples	Mean OD	STDEV	CV%	95% C.I		*p* value	Positive /Negative	Mean Ct	STDEV	CV%	95% C.I		*p* value	Positive /Negative
**LaSota strain**	1.785	0.116	6.548	1.494	2.075	1.0E-5	Positive	16	0.25	1.562	24.717	25.283	0.0002	Positive
**244/14(EC)**	1.402	0.111	7.917	1.126	1.677	3.0E-5	Positive	20	0.1	0.5	19.887	20.113	0.0001	Positive
**289/14(EC)**	1.207	0.088	7.311	0.987	1.426	2.0E-5	Positive	19	0.321	1.694	18.636	19.364	0.0001	Positive
**163/15(EC)**	0.097	0.012	12.876	0.066	0.128	0.01392	Negative	41	0.12	0.292	40.864	41.136	0.01035	Negative
**540/15(ET)**	0.765	0.012	1.584	0.734	0.795	0.0001	Positive	35	0.882	2.521	34.001	35.999	0.004	Positive
**518/15(ET)**	0.72	0.039	5.508	0.621	0.818	1.0E-5	Positive	33	0.173	0.524	32.804	33.196	0	Positive
**546/15(ET)**	1.484	0.004	0.318	1.472	1.496	0	Positive	16	0.346	2.165	15.608	16.392	0.0001	Positive
**79/15(ET)**	1.452	0.05	3.471	1.326	1.577	0.0002	Positive	16	0.173	1.082	15.804	16.196	0	Positive
**538/15(A)**	0.117	0.02	17.907	0.065	0.169	0.01399	Negative	41.5	0.2	0.481	41.274	41.726	0.1036	Negative
**286/15(ET)**	1.029	0.03	2.974	0.953	1.105	0.0002	Positive	30	0.4	1.333	29.547	30.453	0.0007	Positive
**169/15(A)**	1.175	0.029	2.468	1.103	1.247	0.0001	Positive	23	0.655	2.851	22.258	23.742	0	Positive
**534/15(ET)**	1.291	0.04	3.124	1.190	1.391	0.0002	Positive	20	0.7	3.5	19.208	20.792	0	Positive
**556/15(EC)**	0.825	0.095	11.566	0.588	1.062	0.00016	Positive	34	0.3	0.882	33.661	34.339	1.0E-5	Positive
**50/16(K+Li)**	0.085	0.01	11.764	0.060	0.109	0.03444	Negative	43	0.264	0.615	42.701	43.299	0.01795	Negative
**65/16(K+T)**	0.098	0.015	15.433	0.060	0.136	0.02236	Negative	41.8	0.2	0.478	41.574	42.026	0.4481	Negative
**100/16(K+T)**	0.962	0.029	3.025	0.889	1.034	0.0002	Positive	30	0.781	2.603	29.116	30.884	2.0E-5	Positive
**003/16(K+T)**	0.073	0.009	13.067	0.049	0.096	0.29206	Negative	41.666	0.288	0.692	41.34	41.993	0.27944	Negative
**29/16(K+T)**	0.086	0.014	17.364	0.048	0.123	0.08532	Negative	41.533	0.503	1.211	40.964	42.103	0.26175	Negative
**64/16(EC)**	0.074	0.005	7.150	0.06	0.087	0.08498	Negative	43	0.2	0.465	42.774	43.226	0.01369	Negative
**92/17(ET)**	0.067	0.002	3.948	0.060	0.073	1	Negative	42	0.458	1.091	41.481	42.519	1	Negative
**134/17(K+T)**	1.174	0.02	1.703	1.124	1.223	0.0001	Positive	30.666	0.416	1.357	30.196	31.138	0.0008	Positive
**148/17(L+T)**	1.178	0.004	0.339	1.168	1.187	0	Positive	26	0.264	1.017	25.701	26.299	0.0002	Positive
**174/17(L+T)**	1.154	0.007	0.606	1.136	1.171	0	Positive	25	0.3	1.2	24.661	25.339	0.0002	Positive
**31/17(L+T)**	0.091	0.009	9.890	0.068	0.113	0.0103	Negative	43.5	0.556	1.279	42.87	44.13	0.0173	Negative
**81/17(L+T)**	0.094	0.014	15.342	0.058	0.129	0.03015	Negative	40.666	0.493	1.213	40.108	41.225	0.0539	Negative
**83/17(L)**	0.089	0.014	15.626	0.054	0.124	0.0459	Negative	41.666	0.305	0.733	41.321	42.012	0.2889	Negative
**147/17(L+T)**	0.085	0.009	10.846	0.062	0.108	0.02515	Negative	42.333	0.416	0.983	41.862	42.804	0.35367	Negative
**34/19(EC)**	1.235	0.016	1.295	1.195	1.274	0	Positive	21	0.3	1.428	20.661	21.339	0.0002	Positive
**NC**	0.066	0.003	4.545	0.058	0.073	-	Negative	42	0.360	0.858	41.592	42.408	-	Negative

## Discussion

In recent years, aptamers have been investigated as an alternative means for their bio-affinity and target recognition, which has become one of the promising nano-molecules with great interest for medical needs. The use of aptamers is widely extended, as therapeutic molecules, for the development of biosensor or specific delivery means of active molecules [22]. The aptamers are functional short chains of nucleic acids of 20 to 90 bases. Their folding into a variety of 3D structures offers a large area of antigen recognition, allowing them to be powerful agents for targeting and binding to any type of molecules, ions, or whole cells. Their specific recognition of targets and their high affinity in the range of nano- or sub-nanomolar range [23] make them very powerful tools for bio-recognition. They are often compared to antibodies with desirable and higher properties. They are chemically derivable and controllable, with low immunogenicity and quite high physical stability [24, 25]. Their production is quite inexpensive and reproducible. Another major advantage of aptamers is that they can be generated and selected *in vitro* by SELEX from a DNA library of approximately 10^15^ randomized sequences. SELEX is a robust combinatorial chemical screening *in vitro* method and selection process may extend between two to four weeks, which is significantly short in comparison with generation of specific antibodies (months).

Here, we describe the first study that identifies and characterizes DNA aptamers specific to NDV, a major pathogen for poultry industry. DNA aptamers were chosen rather than RNA ones because of their stability and easier usage in the field. Different SELEX protocols for the generation of DNA aptamers against various targets have previously been reported. One of the described protocols, based on high salt concentration for elution of the binders, and limited number of amplifications of the aptamers that bind to the target on the dot blot was used in this study. This protocol that was first reported by Arnold et al. [17] and later was successfully used in our previous work with some modification [15], allowed generation of aptamers using very few selection rounds in comparison to other protocols, which often require up to 10 rounds of selection [26, 27].

Here, the SELEX was performed using three rounds of selection. The first step was the elution of high affinity aptamers using high concentration of NaCl. The sequential increased concentration of NaCl allows gradual release of the aptamer species starting from those with the weakest to the strongest affinity by disturbing the electrostatic interactions. The SELEX was followed by an immuno-blot test, where the eluted DNA oligonucleotides from the 1.5 M of NaCl was amplified by PCR and used to recognize the virus immobilized on a nitrocellulose membrane. The specific aptamers were extracted from the aptamer-spots and then amplified. This step of amplification, isolation on blot and extraction of specific aptamers was repeated three times to enrich the highly specific aptamers with no requirement for counter SELEX.

During the SELEX procedure, the eluted DNA were amplified using asymmetric PCR. This represented one of the most essential steps in the selection process. If it is not controlled correctly, the amplification of the selected aptamers might give rise to a complete loss of the desired sequences [28], and a failure of the selection process [29]. Different protocols for the optimization of the PCR process have been used [30, 31]. In our approach, the enriched DNA library from the third round of SELEX was sequenced using high-throughput sequencing, and analyzed by FASTAptamer tool. High throughput sequencing was used instead of conventional cloning and sequencing, since high throughput sequencing is a powerful approach allowing identification of higher affinity and specificity of ssDNA aptamers that might otherwise not be discovered through the conventional method [32]. After sequencing, the data were first processed using Ion Proton Suite Software, which performs base calling and quality filtering. After the control quality test, data were secondly processed using simple command line to scan sequences for forward and reverse primers and the insert length. In fact, proper reads with 80 nt, including 20 nt of 5’primer + 40 nt random sequence + 20 nt 3’ primer, were sorted. Concatemeric sequences containing only primer sequence were filtered out leaving 40 nt for downstream analysis, using different packages of FASTAptamer toolkit. This software is open source licensing and designed for non-bio-informatics analyst. FASTAptamer includes a collection of scripts that perfectly perform basic analysis steps for all combinatorial selections, regardless of the technology used for the selection process. Thus, we identified five aptamers, which were analyzed and evaluated for their potential use in the sandwich ELAA as a diagnostic assay. Affinity and specificity tests were performed using dc-ELAA, a variant of enzyme-linked immuno-sorbent assay (ELISA), which has been used to study protein-protein interactions [33]. Dc-ELAA results demonstrated that the five selected aptamers bound selectively to NDV with apparent K_d_ values of 24.33 nM to 85.12 nM.

ELAA technique has previously been used to detect various targets such as thrombin, *M*. *tuberculosis* and cocaine detection [34–36], but to our knowledge, this technique has not been used to detect avian viruses, in particular NDV. The ELAA approach may be conducted under different configurations such as direct, indirect and sandwich detection test. Compared to direct and indirect ELAA, the sandwich ELAA seems to be more accurate and stable, depending on the capture aptamers’ efficiency for target recognition, but not on the immobilization, the purity or the concentration of the target [37].

The performance of ELAA has previously been shown to be comparable to that of ELISA technique, and it has been reported as a powerful tool for the diagnostic of viral diseases such as Zika, bovine Parainfluenza and influenza subtype A [38–40].

In our work, the sandwich ELAA test was developed to allow detection of NDV in complex matrices without the need for any purification steps nor sample preparation. Such approach has also been used in other studies to successfully detect, for instance, thrombin, *M*. *tuberculosis* and cocaine with greater performances as compared to assays utilizing antibodies [41, 42].

The sandwich ELAA was initially evaluated using LaSota vaccine strain and total NDV antigen from farm samples. Optimal conditions, including time and temperature of incubation, as well as buffer composition and aptamer concentration were established, allowing the best detection efficiency. Additionally, we found that the use of a combination of Apt_NDV01 and Apt_NDV03 in a sandwich ELAA resulted in the best performing assay to detect and measure different levels of NDV with significant signals over a very low background.

Furthermore, the ELAA based on Apt_NDV01 and Apt_NDV03 aptamers showed high specificity for NDV, and we did not observed any false positive signals when the assay was tested on other live avian viruses such as H120-IBV, IBDV, 1133 reovirus vaccine strains as well as avian influenza isolate -H9N2. However, further validation on larger sample cohorts and other viral agents may be needed.

The obtained results using the sandwich ELAA, when a total of 27 field samples were analyzed, perfectly correlated with the results from current golden standard qRT-PCR method.

The advantages of the sandwich ELAA are the rapid and reproducible production of synthetic aptamers as probes instead of antibodies, and the lack of requirement for sample preparation and viral genome extraction.

In summary, we report the great performance of the combination of SELEX and high throughput sequencing for rapid screening and characterization of aptamers. DNA aptamers targeting NDV were successfully selected after three rounds of evolved enrichment, using LaSota vaccine as a target along with a SELEX process. FASTAptamer toolkit was used to analyse the results obtained with the five selected aptamers. Binding analysis revealed that selected aptamers recognize NDV with high specificity and with nano-molar affinities. The present study is the first report that uses validated novel aptamer-based sandwich detection method to detect NDV in field samples.

## Supporting information

S1 File (PDF)

## References

[1] Alexander D.J., Newcastle disease and other avian paramyxoviruses. Rev Sci Tech, 2000 19(2): p. 443–62. 10.20506/rst.19.2.1231 10935273

[2] Brown C., King D.J., and Seal B.S., Pathogenesis of Newcastle disease in chickens experimentally infected with viruses of different virulence. Vet Pathol, 1999 36(2): p. 125–32. 10.1354/vp.36-2-125 10098640

[3] Mohamed M.H., et al, Sequence analysis of fusion protein gene of Newcastle disease virus isolated from outbreaks in Egypt during 2006. Virol J, 2011 8: p. 237 10.1186/1743-422X-8-237 21592379 PMC3114776

[4] Abenes G., Kida H., and Yanagawa R., Antigenic mapping and functional analysis of the F protein of Newcastle disease virus using monoclonal antibodies. Arch Virol, 1986 90(1–2): p. 97–110. 10.1007/BF01314148 2425781

[5] Peeters B.P., et al, Rescue of Newcastle disease virus from cloned cDNA: evidence that cleavability of the fusion protein is a major determinant for virulence. J Virol, 1999 73(6): p. 5001–9. 10.1128/JVI.73.6.5001-5009.1999 10233962 PMC112544

[6] Absalon A.E., et al, Epidemiology, control, and prevention of Newcastle disease in endemic regions: Latin America. Trop Anim Health Prod, 2019. 10.1007/s11250-019-01843-z PMC6520322 30877525

[7] Haji-Abdolvahab H., et al, Prevalence of avian influenza, Newcastle disease, and infectious bronchitis viruses in broiler flocks infected with multifactorial respiratory diseases in Iran, 2015–2016. Trop Anim Health Prod, 2019 51(3): p. 689–695. 10.1007/s11250-018-1743-z 30377950 PMC7088748

[8] Wang L., et al, Selection of aptamers against pathogenic bacteria and their diagnostics application. World J Microbiol Biotechnol, 2018 34(10): p. 149 10.1007/s11274-018-2528-2 30220026

[9] Tuerk C. and Gold L., Systematic evolution of ligands by exponential enrichment: RNA ligands to bacteriophage T4 DNA polymerase. Science, 1990 249(4968): p. 505–10. 10.1126/science.2200121 2200121

[10] Khoshbin Z., et al, Aptasensors as the future of antibiotics test kits-a case study of the aptamer application in the chloramphenicol detection. Biosens Bioelectron, 2018 122: p. 263–283. 10.1016/j.bios.2018.09.060 30268964

[11] Cimadamore A., et al, New Prostate Cancer Targets for Diagnosis, Imaging, and Therapy: Focus on Prostate-Specific Membrane Antigen. Front Oncol, 2018 8: p. 653 10.3389/fonc.2018.00653 30622933 PMC6308151

[12] Xu J., et al, [Screening of nucleic acid aptamer of lung cancer cells based on cell exponential enrichment ligand system evolution and its application in tumor diagnosis and treatment]. Sheng Wu Yi Xue Gong Cheng Xue Za Zhi, 2018 35(6): p. 964–969. 10.7507/1001-5515.201806006 30583324 PMC9935205

[13] Su L.C., et al, Rapid and highly sensitive method for influenza A (H1N1) virus detection. Anal Chem, 2012 84(9): p. 3914–20. 10.1021/ac3002947 22401570

[14] Fu Y., et al, Exploiting enzyme catalysis in ultra-low ion strength media for impedance biosensing of avian influenza virus using a bare interdigitated electrode. Anal Chem, 2014 86(4): p. 1965–71. 10.1021/ac402550f 24180352

[15] Hmila I., et al, A novel method for detection of H9N2 influenza viruses by an aptamer-real time-PCR. J Virol Methods, 2017 243: p. 83–91. 10.1016/j.jviromet.2017.01.024 28159667

[16] Asha K., et al, Advancements in Nucleic Acid Based Therapeutics against Respiratory Viral Infections. J Clin Med, 2018 8(1). 10.3390/jcm8010006 PMC6351902 30577479

[17] Arnold S., et al, One round of SELEX for the generation of DNA aptamers directed against KLK6. Biol Chem, 2012 393(5): p. 343–53. 10.1515/hsz-2011-0253 22505517

[18] Lamont E.A., et al, A combined enrichment and aptamer pulldown assay for Francisella tularensis detection in food and environmental matrices. PLoS One, 2014 9(12): p. e114622 10.1371/journal.pone.0114622 25536105 PMC4275185

[19] Alam K.K., Chang J.L., and Burke D.H., FASTAptamer: A Bioinformatic Toolkit for High-throughput Sequence Analysis of Combinatorial Selections. Mol Ther Nucleic Acids, 2015 4: p. e230 10.1038/mtna.2015.4 25734917 PMC4354339

[20] Wise M.G., et al, Development of a real-time reverse-transcription PCR for detection of newcastle disease virus RNA in clinical samples. J Clin Microbiol, 2004 42(1): p. 329–38. 10.1128/jcm.42.1.329-338.2004 14715773 PMC321685

[21] Armbruster D.A. and Pry T., Limit of blank, limit of detection and limit of quantitation. Clin Biochem Rev, 2008 29 Suppl 1: p. S49–52. 18852857 PMC2556583

[22] Cai S., et al, Investigations on the interface of nucleic acid aptamers and binding targets. Analyst, 2018 143(22): p. 5317–5338. 10.1039/c8an01467a 30357118

[23] Kikuchi K., et al, Increased inhibitory ability of conjugated RNA aptamers against the HCV IRES. Biochem Biophys Res Commun, 2009 386(1): p. 118–23. 10.1016/j.bbrc.2009.05.135 19501043

[24] Chelliserrykattil J. and Ellington A.D., Evolution of a T7 RNA polymerase variant that transcribes 2'-O-methyl RNA. Nat Biotechnol, 2004 22(9): p. 1155–60. 10.1038/nbt1001 15300257

[25] Kato Y., et al, New NTP analogs: the synthesis of 4'-thioUTP and 4'-thioCTP and their utility for SELEX. Nucleic Acids Res, 2005 33(9): p. 2942–51. 10.1093/nar/gki578 15914669 PMC1140078

[26] Gu H., et al, Magnetic Separation-Based Multiple SELEX for Effectively Selecting Aptamers against Saxitoxin, Domoic Acid, and Tetrodotoxin. J Agric Food Chem, 2018 66(37): p. 9801–9809. 10.1021/acs.jafc.8b02771 30153406

[27] Li W., et al, [Selection of DNA aptamers to cervical intraepithelial neoplasia by SELEX]. Sheng Wu Gong Cheng Xue Bao, 2018 34(5): p. 785–793. 10.13345/j.cjb.170405 29893086

[28] Musheev M.U. and Krylov S.N., Selection of aptamers by systematic evolution of ligands by exponential enrichment: addressing the polymerase chain reaction issue. Anal Chim Acta, 2006 564(1): p. 91–6. 10.1016/j.aca.2005.09.069 17723366

[29] Shao K., et al, Emulsion PCR: a high efficient way of PCR amplification of random DNA libraries in aptamer selection. PLoS One, 2011 6(9): p. e24910 10.1371/journal.pone.0024910 21949784 PMC3174225

[30] Li Y., et al, Development and validation of a new PCR optimization method by combining experimental design and artificial neural network. Appl Biochem Biotechnol, 2010 160(1): p. 269–79. 10.1007/s12010-009-8581-4 19266318

[31] Roux K.H., Optimization and troubleshooting in PCR. Cold Spring Harb Protoc, 2009 2009(4): p. pdb ip66 10.1101/pdb.ip66 20147122

[32] Cho M., et al, Quantitative selection of DNA aptamers through microfluidic selection and high-throughput sequencing. Proc Natl Acad Sci U S A, 2010 107(35): p. 15373–8. 10.1073/pnas.1009331107 20705898 PMC2932614

[33] Hu L., et al, Selection, Characterization and Interaction Studies of a DNA Aptamer for the Detection of Bifidobacterium bifidum. Int J Mol Sci, 2017 18(5). 10.3390/ijms18050883 PMC5454810 28441340

[34] Aimaiti R., et al, Identification and application of ssDNA aptamers against H(3)(7)Rv in the detection of Mycobacterium tuberculosis. Appl Microbiol Biotechnol, 2015 99(21): p. 9073–83. 10.1007/s00253-015-6815-7 26194558

[35] Nie J., et al, A self-assemble aptamer fragment/target complex based high-throughput colorimetric aptasensor using enzyme linked aptamer assay. Talanta, 2013 106: p. 309–14. 10.1016/j.talanta.2012.11.018 23598133

[36] Park J.H., et al, A colorimetric sandwich-type assay for sensitive thrombin detection based on enzyme-linked aptamer assay. Anal Biochem, 2014 462: p. 10–2. 10.1016/j.ab.2014.05.015 24937288

[37] Toh S.Y., et al, Aptamers as a replacement for antibodies in enzyme-linked immunosorbent assay. Biosens Bioelectron, 2015 64: p. 392–403. 10.1016/j.bios.2014.09.026 25278480

[38] Cheng J., et al, Screening and Identification of ssDNA Aptamers against HN Protein for Detection of Bovine Parainfluenza Virus Type 3 Antibodies in Serum. Curr Pharm Biotechnol, 2018 19(11): p. 896–901. 10.2174/1389201019666181031154046 30381069

[39] Lee J.M., et al, An Aptamer-Based Electrochemical Sensor That Can Distinguish Influenza Virus Subtype H1 from H5. J Microbiol Biotechnol, 2017 27(11): p. 2037–2043. 10.4014/jmb.1708.08015 28910866

[40] Shiratori I., et al, Selection of DNA aptamers that bind to influenza A viruses with high affinity and broad subtype specificity. Biochem Biophys Res Commun, 2014 443(1): p. 37–41. 10.1016/j.bbrc.2013.11.041 24269231

[41] Baldrich E., et al, Displacement enzyme linked aptamer assay. Anal Chem, 2005 77(15): p. 4774–84. 10.1021/ac0502450 16053288

[42] Drolet D.W., Moon-McDermott L., and Romig T.S., An enzyme-linked oligonucleotide assay. Nat Biotechnol, 1996 14(8): p. 1021–5. 10.1038/nbt0896-1021 9631044

