# Supplementary material for: Generation of ssDNA aptamers as diagnostic tool for Newcastle avian virus
Source: PLoS One. 2020 Aug 13;15(8):e0237253. doi: 10.1371/journal.pone.0237253 (PMC7425888; doi:10.1371/journal.pone.0237253)
Supplement: S1 File — (PDF) [file pone.0237253.s001.pdf]

Generation of ssDNA aptamers as diagnostic tool for Newcastle avian virus

Boutheina Marnissi[1], Masood Kamali-Moghaddam[2], Abdeljelil Ghram[1] and Issam Hmila[1*]

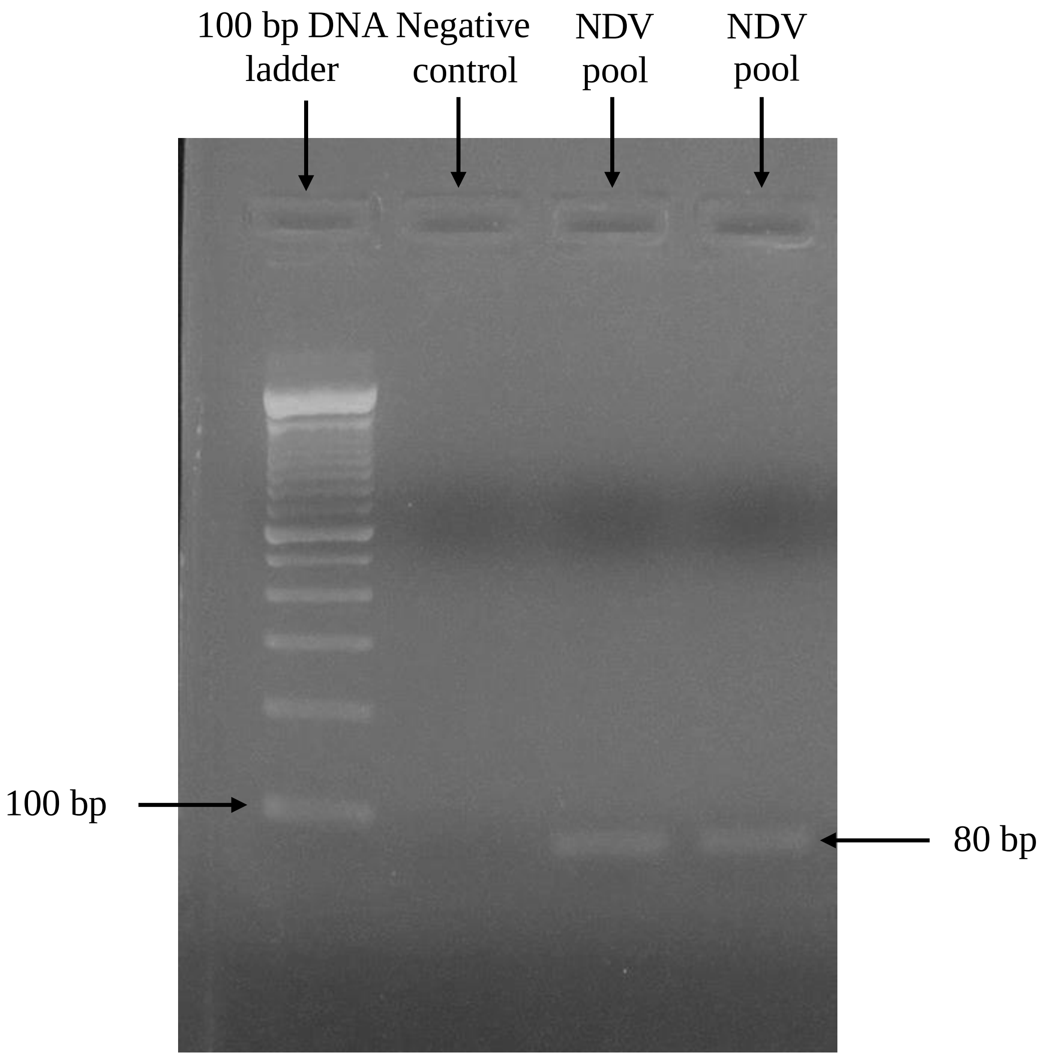

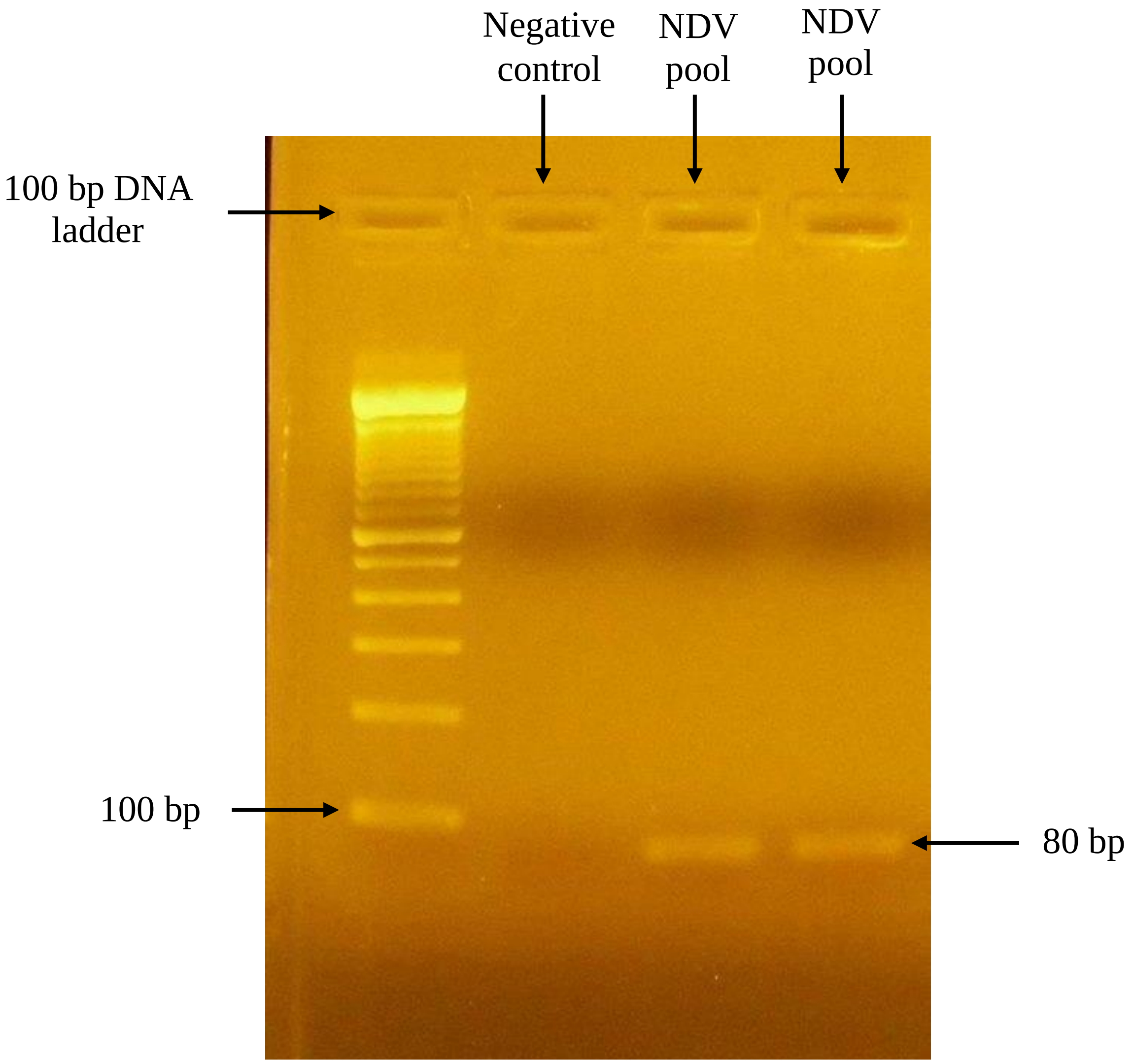

S1 Fig. Symmetric PCR. 2% agarose gel electrophoresis of amplified symmetric PCR product used for high-throughput sequencing of DNA-Aptamers.

Generation of ssDNA aptamers as diagnostic tool for Newcastle avian virus

Boutheina Marnissi[1], Masood Kamali-Moghaddam[2], Abdeljelil Ghram[1] and Issam Hmila[1*]

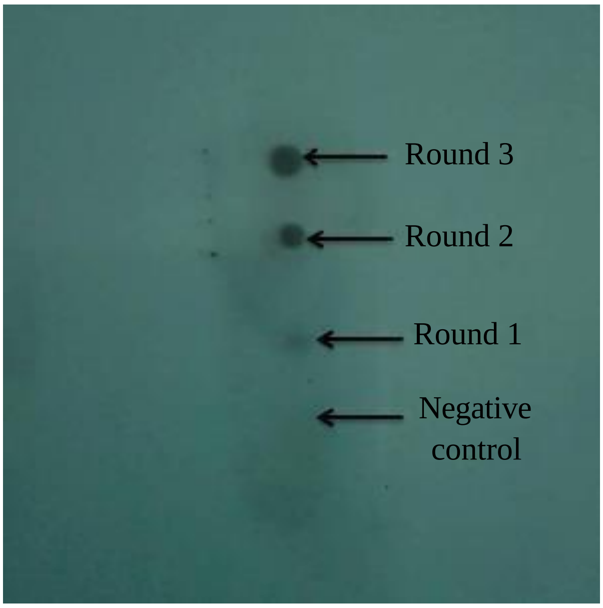

S2 Fig. Immuno-blotting. The fluorescence signal increased after three rounds of aptamer selection

Generation of ssDNA aptamers as diagnostic tool for Newcastle avian virus

Boutheina Marnissi[1], Masood Kamali-Moghaddam[2], Abdeljelil Ghram[1] and Issam Hmila[1*]

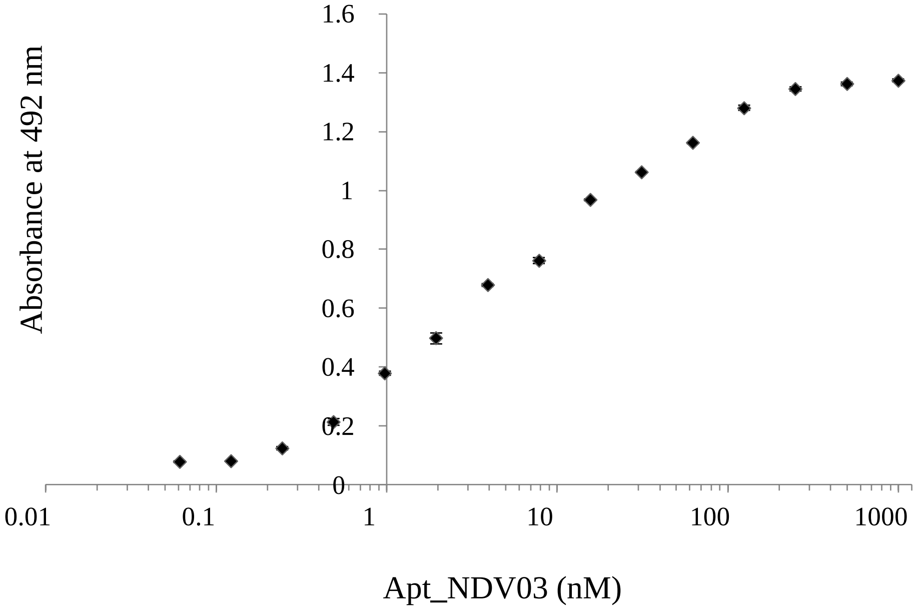

S3 Fig. The compatibility of the selected aptamers in sandwich ELAA. 1 µl of diluted virus was immobilized via biotinylated Apt_NDV03 and an increasing amount of digoxygenin Apt_NDV01 was used to detect the virus. The results show that the detection curve with increasing amount of Apt_NDV01 reach a plateau, indicating that Apt_NDV01 and Apt_NDV03 do not compete.

Generation of ssDNA aptamers as diagnostic tool for Newcastle avian virus

Boutheina Marnissi[1], Masood Kamali-Moghaddam[2], Abdeljelil Ghram[1] and Issam Hmila[1*]

S4 Fig. Secondary structure of ssDNA aptamers. (A) Secondary structure of Apt_NDV01; (B) Secondary structure of Apt_NDV03, using the mfold software.

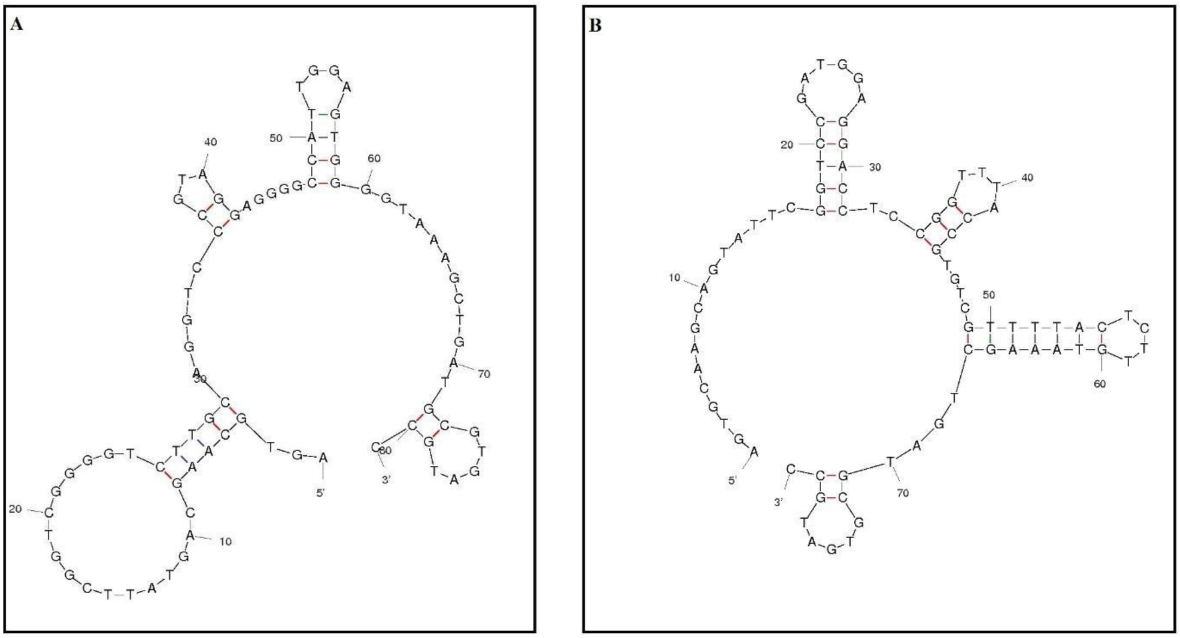

Generation of ssDNA aptamers as diagnostic tool for Newcastle avian virus

Boutheina Marnissi[1], Masood Kamali-Moghaddam[2], Abdeljelil Ghram[1] and Issam Hmila[1*]

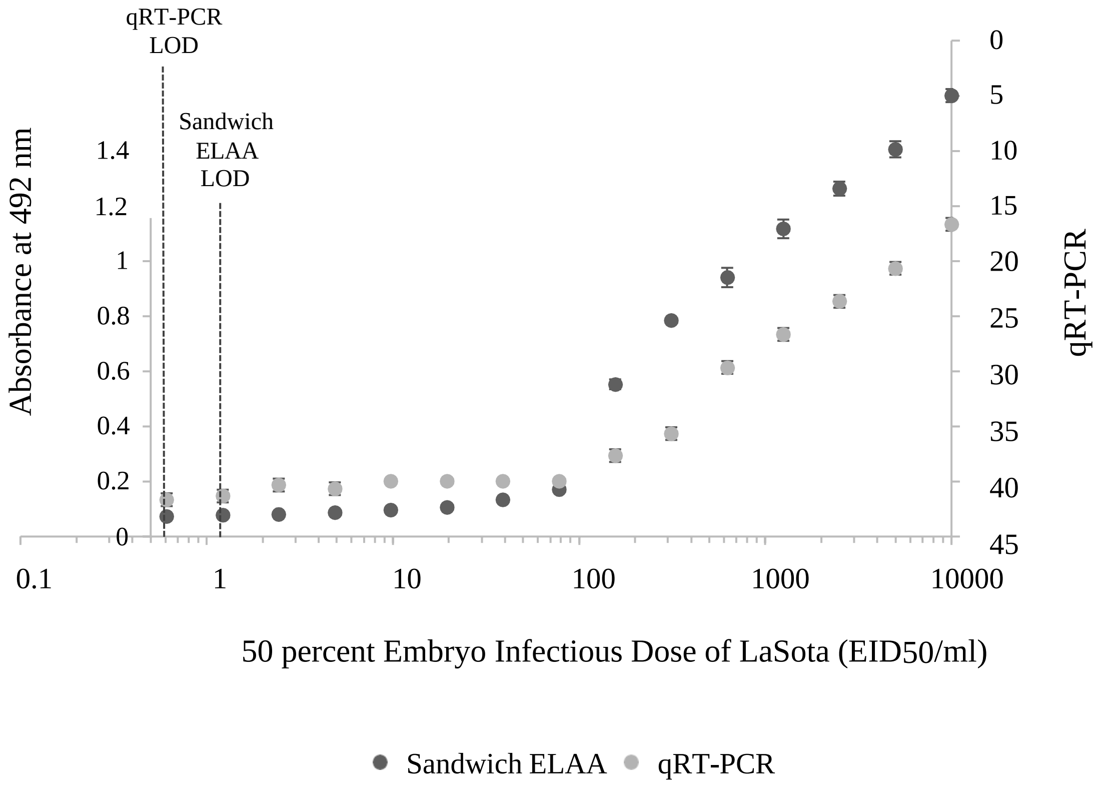

S5 Fig. Comparison between qRT-PCR (grey) and sandwich ELAA (black) for the measurement of LaSota vaccine strain. The right y-axis represents Ct-values for the qRT-PCR test, while the left y-axis represents OD at 492 nm for sandwich ELAA. The x-axis represents the 50 percent embryo infectious dose of LaSota (EID50/ml). All measurements were performed in triplicates. Standard deviations of the mean are indicated with the error bars.

Generation of ssDNA aptamers as diagnostic tool for Newcastle avian virus

Boutheina Marnissi[1], Masood Kamali-Moghaddam[2], Abdeljelil Ghram[1] and Issam Hmila[1*]

S1 Table. The list of samples and their origin

Sample	Region	Veterinary/Farm/Company name	Production	Age
244/14(EC)	Ariana	Mr. Moncef Daly/CRDA	Broilers	3w.
289/14(EC)	Ben Arous	Dr. Hela Memi/CRDA	Broilers	5w.
163/15(EC)	Jendouba	Mr. Samir Albachi/CRDA	Broilers	7w.
540/15(ET)	Sfax	Mr. Karim mezgheni/CRDA	Layers	70w.
518/15(ET)	Sfax	Mr. Mohamed Bouhkris/CRDA	Layers	38w.
546/15(ET)	Sfax	Mr. Nouri Zghal/CRDA	Layers	42w.
79/15(ET)	Sfax	Dr. Hatem Haj Kacem /CRDA	layers	50w.
538/15(A)	Sfax	Mr. Mohamed Boukhris/CRDA	Layers	42w.
286/15(ET)	Ariana	Mr. Moncef Daly/CRDA	Broilers	41d.
169/15(A)	Jendouba	Mr. Kahouli Med Hedi/CRDA	Layers	36w.
534/15(ET)	Sfax	Mr. Nouri Zghal/CRDA	Layers	68w.
556/15(EC)	Sfax	Mr. Adel Rekik/CRDA	Layers	80w.
50/16(K+Li)	Sfax	Dr. Hatem Haj Kacem /CRDA	Layer Breeders	44w.
65/16(K+T)	Beja	Dr. Moez Zmerli /CRDA	Broiler Breeders	76w.
100/16(K+T)	Beja	Dr. Moez Zmerli /CRDA	Broilers	4w.
003/16(K+T)	Ariana	Dr. Kamel Ben Hamida / CRDA	Broiler Breeders	29w.
29/16(K+T)	Beja	Dr. Moez Zmerli /CRDA	Turkey Meat	3w.
64/16(EC)	Beja	Dr. Moez Zmerli /CRDA	Layers	77w.
92/17(ET)	Sidi Bouzid	Dr. Afef Issaoui /CRDA	Broilers	5w.
134/17(K+T)	Nabeul	Dr. Imed Choura/CRDA/Chouihi company	Broilers	5w.
148/17(L+T)	Nabeul	Dr. Imed Choura/CRDA/Sabra company	Broilers	7w.
174/17(L+T)	Nabeul	Dr. Imed Choura CRDA/Chouihi company	Broilers	5w.
31/17(L+T)	Nabeul	Dr. Imed Choura CRDA/ El Marj company	Broilers	3w.
81/17(L+T)	Ben Arous	Dr. Imed Choura CRDA/SNA company	Layers	80w.
83/17(L)	Nabeul	Dr. Lotfi Bennani /CRDA/Chouihi	Layers	52w.

		company		
147/17(L+T)	Nabeul	Dr. Hatem Laamiri / CRDA/Chouihi company	Broilers	4w.
34/19(EC)	Bizerte	Dr. Mohamed Zouari/CRDA	Layers	36w.

CRDA : Regional Departement of Agriculture Development (Ministry of Agricultureof Tunisia)

w : Week

d : day

SNA : Feed company

Generation of ssDNA aptamers as diagnostic tool for Newcastle avian virus

Boutheina Marnissi[1], Masood Kamali-Moghaddam[2], Abdeljelil Ghram[1] and Issam Hmila[1*]

S2 Table. Sample tab-delimited output from high throughput sequencing, sorted to show the full sequences from NDV pool

**>ID**<br>Full sequence
**>9A40N:01334:11618**<br>TAAAGCTGATGCGTGATGCCCAGGGCCTGCTGATCCCACGCCCGCAGCACGTCC<br>GGACTCGACCGAATACTGCTTGCACT
**>9A40N:01334:11622**<br>AGTGCAAGCAGTATTCGGTCAATCTTGCATATACATTCATAGTTTACTCGCTGGTT<br>GATGGGCATCACGCATCAGCTTTA
**>9A40N:01334:11627**<br>AGTGCAAGCAGTATTCGGTCGTCTGTATTTGAATGCCTTCCCCACTTTTCATCGTG<br>TACTGGCATCACGCATCAGCTTTA
**>9A40N:01334:11634**<br>AGTGCAAGCAGTATTCGGTCCCTCGGTCTCTGAAGGCCCGGGTGGAACTGGTCG<br>TCCGGTGGCATCACGCATCAGCTTTA
**>9A40N:01335:11596**<br>AGTGCAAGCAGTATTCGGTCGACTTAGTCTGGCATTGGGCTCGGGTCGGACGAC<br>GGAGGGGGCATCACGCATCAGCTTTA
**>9A40N:01335:11597**<br>TAAAGCTGATGCGTGATGCCGTACCGACTTGTCCCATGCTACTGGACGCATTAGC<br>CCGACCGAATACTGCTTGCACT
**>9A40N:01335:11614**<br>TAAAGCTGATGCGTGATGCCTGAGACATGAGAGGGAGGGATAGGGATACGAGGA<br>ATGGAAGACCGAATACTGCTTGCACT
**>9A40N:01335:11623**<br>TAAAGCTGATGCGTGATGCCTATCATATAGCATAGCTCTTAAAATTGGGCAGTTGT<br>ATCTGACCGAATACTGCTTGCACT
**>9A40N:01336:11600**<br>TAAAGCTGATGCGTGATGCCCCTTCAGCCACCTACTTAGACTCGGGGAGGCCAA<br>CCCATTGACCGAATACTGCTTGCACT
**>9A40N:01336:11594**<br>AGTGCAAGCAGTATTCGGTCACCGGTCGAGTGTTTCTCGTCATTCTCGGATCCTA<br>AGCACGGCATCACGCATCAGCTTTA

Generation of ssDNA aptamers as diagnostic tool for Newcastle avian virus

Boutheina Marnissi[1], Masood Kamali-Moghaddam[2], Abdeljelil Ghram[1] and Issam Hmila[1*]

S3 Table. Sample tab-delimited output from FASTAptamer-Count, sorted to show the most highly enriched sequences from NDV pool

**>Rank-Reads-RPM**	Sequence
**>1-7620-1362.29**	GGGGTCTTGCAGGTCCCGTAGGAGGGGCCATTGGAGTGGGG
**>2-4235-757.13**	AACTTATCGGAGCGTGATTTCCGTCTCGCCGCTTTCCTTT
**>3-3262-583.17**	CGATGGAGGACCTCCGGTTTACCGTGTCGTTTTACTCTTG
**>4-2769-495.04**	CCTCGCTATGATGGAGTGCGTTTAGATCAGGGAACGGGTT
**>5-2657-475.01**	CCTACGTTGGAGTGGGGTTTGCGCAGGCCGTTCTTTCCAA
**>6-2203-393.85**	TGCCGTTTGTCACAGTACTGTGGGTCATGACGTTGCACTT
**>7-2184-390.45**	TTGGACTAAGCTGATTACACACTCCATGAGTGTCTCCACC
**>8-2155-385.27**	TACAATCCCCGAAGGAGGGGTAAATCTTAGTGTTGTACCG
**>9-2011-359.52**	GCCCGTGGTGTGAGGGTGATACCTCCCTGCTGCTTTACCT
**>10-1795-320.91**	GGGGTTAGTGGTAATGCTTTGGGCACGGTGTCCTGTACGT

Generation of ssDNA aptamers as diagnostic tool for Newcastle avian virus

Boutheina Marnissi[1], Masood Kamali-Moghaddam[2], Abdeljelil Ghram[1] and Issam Hmila[1*]

S4 Table. Sample tab-delimited output from FASTAptamer-Cluster, sorted to show the most highly enriched sequences per cluster from NDV pool

**>Rank-Reads-RPM-Cluster-Rank in Cluster- Levenshtein Edit distance**<br>Sequence
**>1-7620-1362.29-1-1-0**<br>GGGGTCTTGCAGGTCCCGTAGGAGGGGCCATTGGAGTGGGG<br>**>27-613-109.59-1-2-1**<br>GGGGTCTTGCAGGTCCCGTAGGAGGGGCCATTGGAGTGGG
**>2-4235-757.13-2-1-0**<br>AACTTATCGGAGCGTGATTTCCGTCTCGCCGCTTTCCTTT<br>**>47-316-56.49-2-2-**1<br>AACTTATCGGAGCGTGATTTCCGTCTCGCCGCTTTCCCTT
**>3-3262-583.17-3-1-0**<br>CGATGGAGGACCTCCGGTTTACCGTGTCGTTTTACTCTTG
**>4-2769-495.04-4-1-0**<br>CCTCGCTATGATGGAGTGCGTTTAGATCAGGGAACGGGTT
**>5-2657-475.01-5-1-0**<br>CCTACGTTGGAGTGGGGTTTGCGCAGGCCGTTCTTTCCAA
**>6-2203-393.85-6-1-0**<br>TGCCGTTTGTCACAGTACTGTGGGTCATGACGTTGCACTT
**>7-2184-390.45-7-1-0**<br>TTGGACTAAGCTGATTACACACTCCATGAGTGTCTCCACC
**>8-2155-385.27-8-1-0**<br>TACAATCCCCGAAGGAGGGGTAAATCTTAGTGTTGTACCG
**>9-2011-359.52-9-1-0**<br>GCCCGTGGTGTGAGGGTGATACCTCCCTGCTGCTTTACCT<br>**>36-404-72.23-9-2-1**<br>GCCGTGGTGTGAGGGTGATACCTCCCTGCTGCTTTACCT
**>10-1795-320.91-10-1-0**<br>GGGGTTAGTGGTAATGCTTTGGGCACGGTGTCCTGTACGT
